# Preparation and characterization of a natural composite scaffold composed of chitosan, hydroxyapatite, and graphene oxide for bone repair

**DOI:** 10.1038/s41598-026-44493-6

**Published:** 2026-03-26

**Authors:** Chunliang Li, Feng Qin, Shouhong Zhao, Qiwei Meng, Qianmei He, Haining Fan

**Affiliations:** 1 Research Center for High Altitude Medicine, Key Laboratory of High Altitude Medicine (Ministry of Education), Key Laboratory of Application and Foundation for High Altitude Medicine Research in Qinghai Province (Qinghai-Utah Joissnt Research Key Lab for High Altitude Medicine), The Research Key Laboratory for Echinococcosis of Qinghai Province, Qinghai University, Xining, China; 2 Qinghai University Affiliated Hospital, Xining, China; 3 Qinghai Provincial People’s Hospital, Xining, China

**Keywords:** Composite materials, Characterization, Mesh structure, Biotechnology, Chemistry, Materials science, Nanoscience and technology

## Abstract

The preparation of biocompatible scaffolds from natural substances for tissue engineering offers a beneficial alternative to synthetic materials for tissue repair. In this study, we prepared and characterized a composite scaffold composed of chitosan (CS), nano-hydroxyapatite (n-Hap), and graphene oxide (GO) for bone repair, and investigated its optimal composition. Fourier transform infrared spectroscopy (FTIR), scanning electron microscopy (SEM), and X-ray diffraction (XRD) test were employed to analyze the individual components and characterize the structure, morphology, and properties of the composite scaffold. The results confirmed the successful integration of the three components, with optimal performance achieved when GO was added at a concentration of 1 wt%. GO adhered to the surface of the CS network, promoting n-Hap adsorption. As the GO content increased, the surface adsorption capacity correspondingly increased, and the scaffold exhibited superior cell growth properties and significantly enhanced compressive strength. This enables load transfer through the GO framework, inducing a nonlinear stress increase from 66.6 MPa to 243.4 MPa. At 60% deformation, the optimal compressive strength reached 53.4 MPa.Meanwhile, the degradation rate decreased linearly with increasing GO content (from 13.1% to 6.1% over 21 days), demonstrating the effectiveness of this barrier effect. This scaffold, particularly the 1.0 wt% GO formulation, demonstrated sound choice for clinical translation as a load-bearing bone defect repair material due to its mechanical strength matching that of cortical bone, a degradation rate compatible with the bone regeneration cycle, and excellent cellular compatibility. The CS/n-HAP/GO composite scaffold developed in this study achieved good performance in mechanical, degradation, and biological properties compared with previously reported similar materials, and has realized synergistic optimization of all three aspects. This formulation of three biocompatible materials provides a promising basis for fundamental bio-scaffold research and potential applications in bone repair.

## Introduction

Bone is a tissue with limited regenerative capacity, and when the defect size exceeds its repair ability, irreversible damage may occur^1,2^. Bone tumors, severe skeletal trauma, or congenital malformations can cause damage beyond the tissue’s capacity for self-healing and the resulting irreversible bone deformities often lead to severe, lifelong disorders that adversely affect the patients’ physical and mental health^3^. Although autologous bone grafting has long been considered the gold standard for bone repair in clinical practice, it is associated with several limitations, including donor site morbidity, infection risk and limited graft availability^4–6^. To address the challenges associated with large bone defects, biocompatible scaffolds have been developed as artificial extracellular matrices to support bone tissue regeneration. Through the rational selection of natural, non-toxic materials and intelligent composite design, three-dimensional (3D) scaffold frameworks with favorable biocompatibility and mechanical properties have been constructed to promote bone tissue growth^7,8^.

The main inorganic component of human and animal bone is hydroxyapatite (Hap, Ca₅(PO₄)₃OH). When introduced into the body, Hap exhibits good biodegradability and ion-exchange capability, gradually releasing Ca²⁺ and PO₄³⁻ ions^9^. These ions participate in bone metabolism and contribute to bone growth and regeneration^10–12^. Furthermore, the porous and loosely packed structure of Hap provides a large specific surface area for interaction with surrounding bone tissue and body fluids. This biomimetic microenvironment facilitates the localized accumulation of Ca²⁺ and PO₄³⁻, as well as other biologically relevant ions, thereby promoting the nucleation and deposition of new calcium phosphate phases and enhancing osteointegration. Recent studies have shown that fluorohydroxyapatite-based composite scaffolds exhibit promising performance in bone tissue engineering applications^13^. For example, Soltani et al.^14,15^ prepared sodium alginate/fluorohydroxyapatite composite scaffolds, which demonstrated excellent cell viability and mechanical properties. In addition, the incorporation of functional ions or nanoparticles has been reported to increase scaffold density and further improve mechanical strength^16^. For instance, Dehghan et al.^17^ introduced silver nanoparticles into glass-ceramic materials during sintering, resulting in denser crystalline structures and enhanced mechanical performance. Nanomaterials have also been shown to promote wound healing and cellular responses. Mahmoudi et al.^18^ incorporated silver nanoparticles into bioactive glass (BG), polyvinyl alcohol (PVA), and curcumin (Cur)-based scaffolds, achieving a cell viability of 93.58 ± 1.36%, demonstrating excellent biocompatibility. Based on these findings, nanoscale hydroxyapatite was selected in this study to improve the performance of the prepared scaffold.

Chitosan (CS) is a biodegradable and biocompatible polysaccharide derived from chitin^19^, which is abundantly available as a byproduct of the shellfish industry, thereby offering environmental and economic advantages^20–23^. Previous studies have demonstrated the feasibility of using CS-based composites for bone tissue engineering. For example, Soltani et al.^24^ prepared multifunctional scaffolds using bioactive glass (BG), aloe vera (AV), and waste quail eggshell (QE) powder, and the resulting starch-BG-AV-QE scaffold exhibited cell viability exceeding 95%, providing valuable insights into scaffold design for bone regeneration. However, CS cannot be used alone for bone repair applications due to its poor stability in acidic environments and limited mechanical strength^25^. Its in vivo stability and functional performance can be improved through acetylation, complexation and other chemical modifications. In addition, the positively charged amino groups on CS chains enable the formation of polyelectrolyte complexes with negatively charged Hap particles in aqueous systems^26^. Consequently, CS and Hap are commonly combined through simple blending followed by freeze-drying to fabricate porous bone scaffold^27^. Nevertheless, the relatively low mechanical strength and rapid degradation rate of CS/Hap composites remain significant limitations.

Graphene oxide (GO) is a novel nanomaterial commonly used for bone repair applications. Its structure contains a large number of oxygen-containing functional groups, such as hydroxyl and carboxyl groups, which endow GO with excellent hydrophilicity and abundant protein adsorption sites^28,29^. These properties facilitate the adsorption of osteogenic factors and enhance cell adhesion in osteogenic environments. The incorporation of GO into CS/Hap composites has been reported to improve mechanical strength and structural stability. For example, Nanda et al.^30^ synthesized CS/GO gels for controlled drug release, while Ye et al.^31^ prepared Fe₃O₄/CS/GO composites using glutaraldehyde, which demonstrated efficient adsorption of specific blood proteins. Qiu et al.^32^ fabricated low-temperature CS/GO composite scaffolds with uniform pore structures and notable antimicrobial activity, highlighting the multifunctional potential of GO-based biomaterials.

In this study, we prepared CS/n-Hap/GO composite scaffolds and investigated the effect of GO content on scaffold performance. The composition and microstructure of the scaffolds were characterized using X-ray photoelectron spectroscopy (XPS) and scanning electron microscopy (SEM), and mechanistic analyses were conducted to elucidate the key factors influencing scaffold performance. The optimal formulation was determined through mechanical testing, porosity evaluation, and swelling behavior analysis. Biocompatibility was assessed in vivo by evaluating scaffold degradation behavior and cell viability. By comparing our experimental results with the latest existing research, we have demonstrated that our experimental approach outperforms these materials in terms of experimental design, comprehensive performance, and microstructure, exhibiting core advantages *in bone-level initial cortical strength and controlled biodegradability. Overall*,* this study identified an optimized CS/n-HAP/GO scaffold composition*,* demonstrating that GO as a nanoscale reinforcing phase enhances the mechanical strength and degradation stability of CS/n-HAP scaffolds*,* which provides* theoretical support for its potential applications in bone tissue engineering and related biomedical fields.

## Materials and methods

### Materials

Chitosan (CS, ≥ 95% deacetylation, Sigma-Aldrich Shanghai Trading Co., Ltd., Shanghai, China), nano-hydroxyapatite (n-Hap, AR, Shanghai Macklin Biochemical Technology Co., Ltd., Shanghai, China), graphene oxide (GO, > 99%, Shanghai Macklin Biochemical Technology Co., Ltd., Shanghai, China), glacial acetic acid (AR, ≥ 98%, Sinotrade Chemical Co., Ltd., Beijing, China), sodium hydroxide (NaOH, AR, Shanghai Macklin Biochemical Technology Co., Ltd., Shanghai, China), anhydrous ethanol (AR, Tianjin Ou Bocai Chemical Co., Ltd., Tianjin, China), phosphate buffered saline (PBS, AR, HyClone, Logan, UT, USA) were used in this study.

### Preparation of CS/n-Hap/GO-x wt% gels

Chitosan (4 g) was dissolved in 200 mL of 0.5 M acetic acid under stirring at 1000 rpm to obtain solution 1. To prepare solution 2, 4 g of n-Hap was added to 200 mL of solution 1 and dispersed by stirring at 1000 rpm for 4 h. Solution 3 was prepared by dispersing 0.1 g of GO in 100 mL of deionized water under stirring at 1000 rpm for 1 h, followed by ultrasonic treatment for 2 h. Subsequently, appropriate volumes of solution 3 were added to solution 2 and stirred at 1000 rpm for 24 h to obtain CS/n-Hap/GO-x wt% gels containing 0, 0.3, 0.5, 0.7, and 1.0 wt% GO. The resulting suspensions were further ultrasonically dispersed for 2–4 h, transferred into 24-well plates at the desired GO content, and lyophilized at − 80 °C for 36 h.

### Preparation of CS/n-Hap/GO-x wt% scaffolds

The prepared CS/n-Hap/GO-x wt% samples were cross-linked in 1 M NaOH for 4 h and then washed 3–5 times with deionized water until the pH was 7. The samples were transferred into 24-well plates and lyophilized at −80 °C for 36 h to obtain the final CS/n-Hap/GO-x wt% composite scaffolds.

### Testing and characterization of the scaffolds

#### Total reflectance Fourier transform infrared (FTIR) spectroscopy

Data were obtained using a Nicolet 6700 infrared spectrometer equipped with a DLaTGS detector, with 32 scans, a resolution of 4 cm⁻¹, and a scanning range of 500–4000 cm⁻¹.

#### X-ray photoelectron spectroscopy (XPS)

The surface layers of scaffold samples were mounted on the XPS sample stage, and elemental composition of the samples was analyzed using an ESCALAB Xi⁺ X-ray photoelectron spectrometer (Thermo Scientific). The survey scan was performed with a scanning step of 1 eV, pass energy of 100 eV, and dwell time of 50 ms, while high-resolution spectra were acquired with a step size of 0.05 eV, pass energy of 20 eV, and dwell time of 100 ms.

#### Scanning electron microscopy

The prepared scaffolds were sputter-coated with gold using a vacuum coater. The micro-morphology of the samples was observed using a JSM-7900 F SEM (JEOL) operated at an accelerating voltage of 10 kV.

#### Mechanical properties test

Compressive strength and strain behavior are key parameters for evaluating mechanical properties. Cylindrical scaffold samples (10 mm diameter × 10 mm height) were prepared for each group. Mechanical testing was conducted using an electronic universal testing machine at a compression rate of 0.6 mm/min and a temperature of 23 °C. The elastic modulus was calculated from the stress–strain curves, and the average of three independent measurements was reported for each group.

####  X-ray diffraction performance analysis

Crystallinity was analyzed using a D-Max 2500 X-ray diffractometer (Rigaku, Japan) with a Cu Kα₁,₂ radiation source. The X-ray wavelength was 0.154 nm, the scanning range was 5–60°, the scanning speed was 5°/min, and the step size was 0.02°. The tube voltage and current were 40 kV and 200 mA, respectively.

#### Measurement of porosity

The specific gravity bottle was weighed and recorded as m_1_. Each scaffold was weighed (m_0_), placed into the bottle, and filled with anhydrous ethanol. After ultrasonic treatment to remove trapped air, the bottle was weighed (m_2_). The scaffold was then removed, and the bottle containing the remaining ethanol was weighed (m_3_). Three parallel measurements were performed for each group, and porosity was calculated using Eq. (1):

1 $$\%porosity=\frac{{m}_{2}-{m}_{3}-{m}_{0}}{{m}_{1}-{m}_{3}}\times100\%$$

#### Swelling ratio test

Each scaffold was weighed (m_0_) and immersed in deionized water for 2 h. Excess surface water was gently removed with filter paper, and the scaffold was weighed again (m_1_). Three parallel measurements were conducted, and the swelling ratio was calculated using Eq. (2):

2 $$\%expansion=\frac{{m}_{1}-{m}_{0}}{{m}_{0}}\times100\%$$

#### In vitro degradation rate of scaffolds

Scaffolds were weighed (m_0_), immersed in sterile PBS, and incubated at 37 °C. Samples were collected at 3, 6, 9, 12, 15, 18, and 21 days, rinsed three times with deionized water, and weighed after freeze-drying (m_1_). The degradation rate was calculated using Eq. (3):

3 $$\%degradation=\frac{{m}_{1}-{m}_{0}}{{m}_{0}}\times100\%$$

#### In vitro cytotoxicity assay

Mouse embryonic osteoblast precursor cells (MC3T3-E1, subclone 14; Prosperity Life Sciences Co.) were used. Cells in the exponential growth phase (passages 5–8) were harvested, suspended, and seeded into 96-well plates at a density of 3 × 10³ cells/well, with five replicates per group. After incubation at 37 °C with 5% CO₂ for 24 h, the medium was replaced with fresh complete medium for the control group, while experimental groups were cultured with scaffold extracts. Cells were cultured for 1, 3, 5, and 7 days, and the medium was replaced every two days. Toxicity grading and growth characteristics are summarized in Table 1.

**Table 1:** Effects of CS/n-Hap/GO-x wt% scaffolds on adhesion and growth of osteoblast precursor cells.

Toxicity	Cellular morphology
Nontoxic	Cells exhibit normal morphology with good adhesion and a fibroblast-like pattern.
Mildly toxic	Cells grow well and are mostly adherent, but with a small number of dead cells floating.
Toxic	Poor cell adhesion is observed, with many dead cells and a necrosis rate of 30% or higher.
Severely toxic	Cells are non-adherent and the percentage of dead cells exceeds 90%.

For the CCK-8 assay, cells were washed twice with PBS, and 100 µL of CCK-8 working solution was added to each well. After incubation at 37 °C for 0.5–1 h in the dark, absorbance at 450 nm was measured using a microplate reader to determine cell viability (Table 2).

**Table 2:** Toxicity grading based on cell viability.

Toxicity grade	Cell viability (%)	Standard of judgment
0	> 100	acceptable
1	75–100	acceptable
2	50–75	toxic
3	25–50	toxic
4	1–25	toxic
5	< 1	toxic

## Results and discussion

### FTIR analysis

**Fig. 1:**
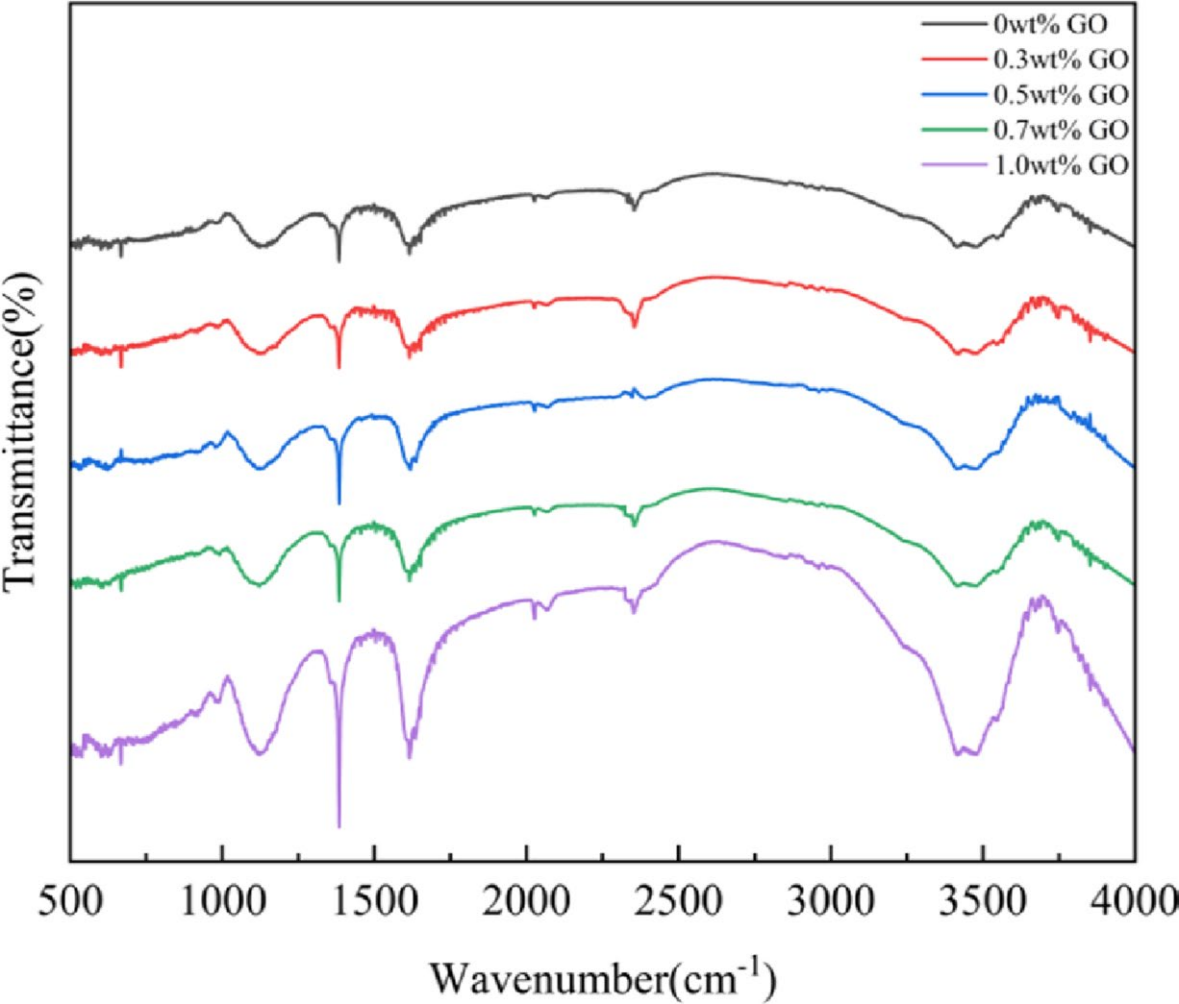
FTIR spectra of CS/n-Hap/GO-x wt% scaffolds with increasing graphene oxide (GO) contents.

As shown in Fig. 1, the O–P–O bending vibration absorption peak at 562 cm⁻¹ and the P–O antisymmetric stretching vibration of n-Hap were shifted to 1100 cm⁻¹, and the changes in these absorption bands demonstrated that the CS/n-Hap composites were successfully prepared. The absorption peaks at 1571 and 1644 cm⁻¹ corresponded to the stretching vibrations of N–H and –C = O in –NHCO–, indicating that the –COOH groups on the surface of GO and the –NH₂ groups in CS underwent an amide reaction to generate –NHCO–, forming strong interfacial amide bonds between CS and GO^33,34^.

The oxygen-containing groups (–OH, –COOH, and –C = O) enriched on the surface of GO can also form hydrogen bonds with –NH₂ and –OH groups in CS, resulting in a shift of the –OH stretching vibration peaks to lower wavenumbers (0 wt% GO: 3493; 0.3 wt% GO: 3489; 0.5 wt% GO: 3485; 0.7 wt% GO: 3486; 1.0 wt% GO: 3479)^35^. Hydrogen bonding reduces the force constants of chemical bonds, resulting in lower energy requirements for molecular stretching vibrations, which is manifested as a decrease in wavenumber in the IR spectra. These results further confirm the successful incorporation of GO into the CS/n-Hap composite.

### X-ray photoelectron spectroscopy (XPS) analysis

**Fig. 2:**
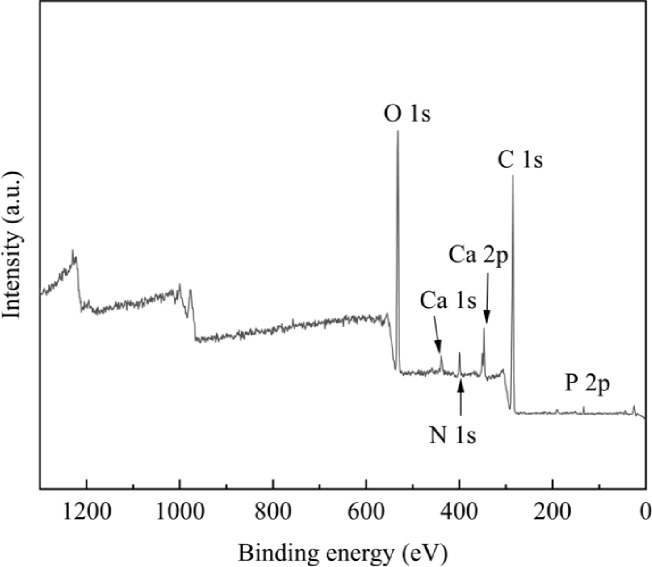
X-ray photoelectron spectroscopy (XPS) elemental analysis of CS/n-Hap/GO-x wt% scaffolds with different GO contents.

To further characterize the different CS/n-Hap/GO-x wt% scaffolds, we performed XPS to determine their elemental composition. Figure 2 shows that the main peak of C1Swas located at 284.8 eV, while peaks at 398.4 eV and 531.5 eV corresponded to N1s and O1s, respectively. The peaks at 132.8 eV and 346.9 eV indicated the presence of P2p and Ca2p, respectively^36^. These results, together with the FT-IR analysis, confirm the successful fabrication of CS/n-Hap/GO-x wt% scaffolds.

### Scanning electron microscopy (SEM) imaging

**Fig. 3:**
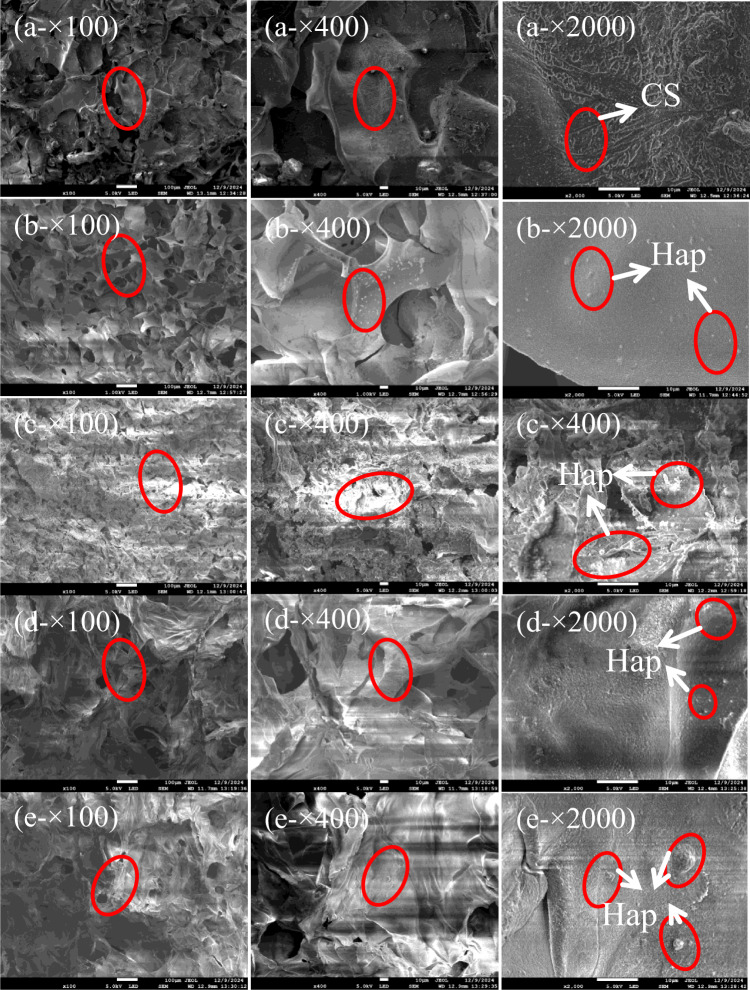
SEM images (100×, 400× and 2000× magnification) of CS/n-Hap/GO-x wt% scaffolds with different GO contents. (**a**) The CS/n-Hap/GO-0 wt% scaffold. (**b**) The CS/n-Hap/GO-0.3 wt% scaffold. (**c**) The CS/n-Hap/GO-0.5 wt% scaffold. (**d**) The CS/n-Hap/GO-0.7 wt% scaffold. (**e**) The CS/n-Hap/GO-1.0 wt% scaffold.

To assess the effect of increasing GO content on scaffold morphology, CS/n-Hap/GO-x wt% scaffolds were examined by SEM at three different magnifications (Fig. 3). At 100× magnification, the scaffold exhibited a three-dimensional mesh structure, provided sufficient space for cell adhesion, proliferation, differentiation and potential formation of new bone matrix. With increasing GO content, the pore size gradually decreased and the structure became denser. This effect was attributed to the formation of cross-links between GO and CS/n-Hap; higher GO content resulted in increased cross-link density, leading to a more compact scaffold structure. The reduced pore size restricted the penetration of water molecules, inhibited ice crystal formation during freeze-drying, and ultimately decreased pore size.

At 2000× magnification, the microstructure was clearly observed, and GO attachment to the CS/n-Hap surface was evident. The thickness of the pore walls increased with increasing GO content, reaching a maximum at 1.0 wt% GO. Thicker pore walls enhanced mechanical strength, helped to prevent structural collapse after implantation in bone defect sites. This observation was consistent with the mechanical performance results described below.

### Mechanical performance tests

**Fig. 4:**
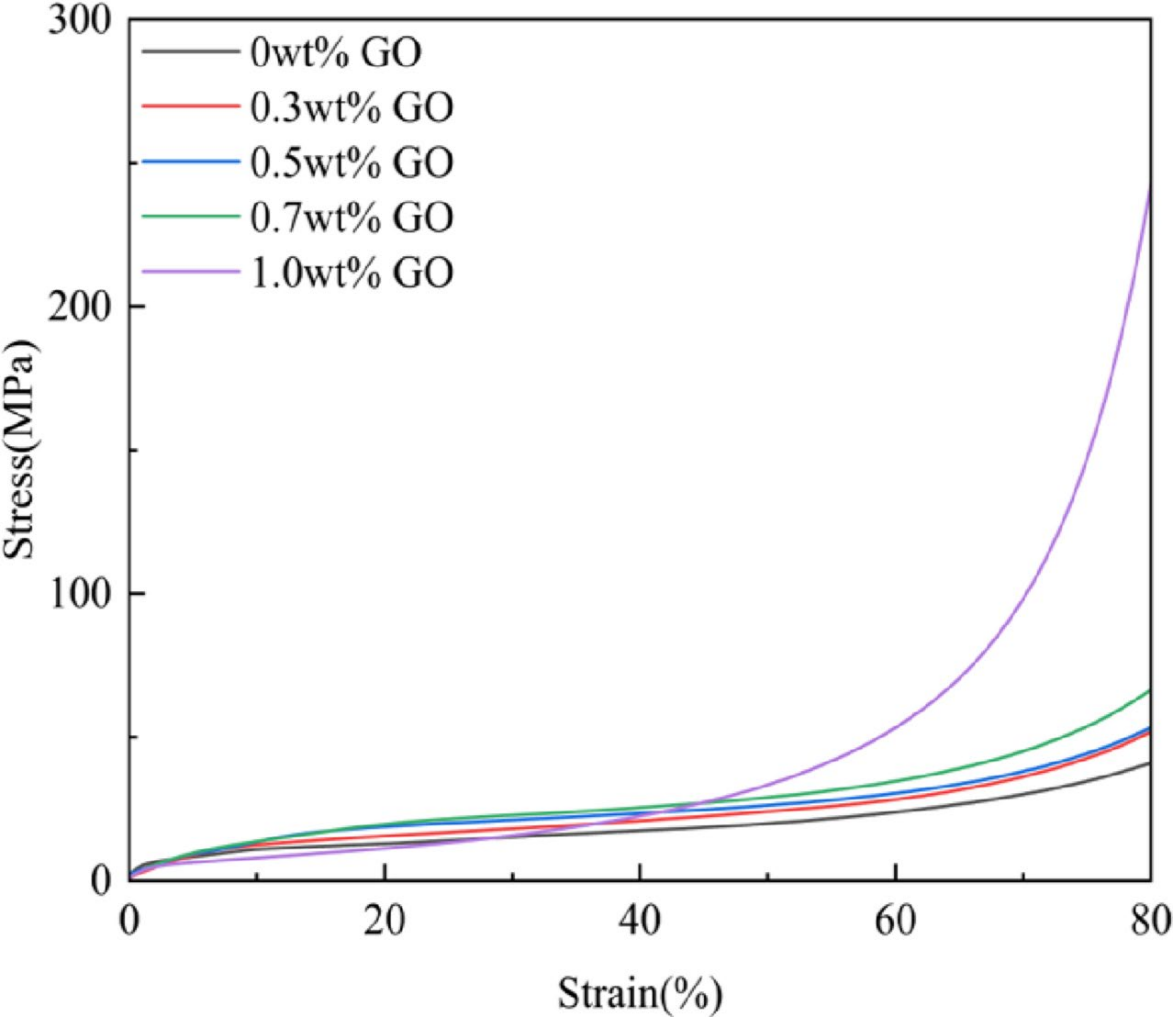
Compressive strength of CS/n-Hap/GO-x wt% scaffolds with increasing GO contents.

**Fig. 5:**
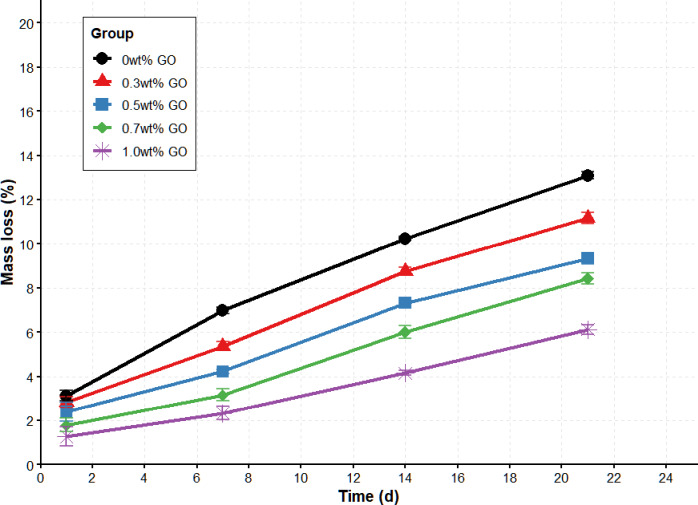
In vitro degradation behavior of CS/n-Hap/GO-x wt% containing different GO contents.

Adequate mechanical strength is essential for bone tissue engineering scaffolds to support load and withstand local stresses. Mechanical properties are influenced by molecular bonding strength, bonding mode, chemical composition, scaffold microstructure, and porosity. Figure 4; Table 3 show that increasing GO content resulted in enhanced compressive strength, which can be attributed to chemical bonding between oxygen-containing functional groups on GO and CS/n-Hap, increased molecular weight, and enhanced intermolecular interactions, leading to a more compact structure. As shown in Table 3, the average data from the three experiments indicate that when the compressive strain reached 80%, the compressive strength of the CS/n-Hap/GO-1.0 wt% scaffold significantly increased to 243.41 MPa. Compared with the mechanical properties of similar scaffolds reported by Yu, Z. et al.^37^, this value represents a 40% improvement. *Overall*,* the addition of GO achieved the “three birds with one stone” effect: firstly*,* mechanical strength was improved (stress increased from 41.03 MPa to 243.41 MPa*,* a roughly 6-fold increase); secondly*,* as shown in* Fig. 5, *degradation stability was enhanced (the 21-day degradation rate decreased from 13.09% to 6.13%*,* a reduction of over 50%); and finally*,* structural strain remained stable (strain in all groups was maintained at ~ 80%*,* indicating that the material’s toughness was not compromised)*. Thus, increasing GO content promoted greater attachment of CS/n-Hap, further enhancing mechanical performance. In addition, SEM observations confirmed that 1.0 wt% GO increased pore wall thickness, contributing to improved mechanical strength.

**Table 3:** Mechanical properties of CS/n-Hap/GO-x wt% scaffolds.

Bracket Sample	Strain (%)	Stress (MPa)
CS/n-Hap/GO-0wt%	80.02 ± 0.44%	41.03 ± 1.37
CS/n-Hap/GO-0.3wt%	80.05 ± 0.34%	51.95 ± 1.41
CS/n-Hap/GO-0.5wt%	80.01 ± 0.25%	53.43 ± 0.95
CS/n-Hap/GO-0.7wt%	80.07 ± 0.41%	66.57 ± 1.16
CS/n-Hap/GO-1.0wt%	80.03 ± 0.37%	243.41 ± 1.72
Standard deviation(F)	0.028	12107.031
Significant level(P)	0.998	<0.01

Note: Data are shown as means ± SD, *P* < 0.1.

*The introduction of GO resulted in quantitative develop in the scaffold’s mechanical properties and enhanced the compressive strength in a dose-dependent manner. Compared to the control group without GO (0 wt% GO)*,* the addition of 1.0 wt% GO increased the compressive stress from (41.03 ± 1.37) MPa to (243.41 ± 1.72) MPa*,* representing a remarkable 493% increase (p < 0.001). This indicates that the addition of just 1.0 wt% GO elevated the material’s load-bearing capacity by approximately 5.9-fold*,* reaching the mechanical range of natural cortical bone (100–230 MPa). Notably*,* the fracture strain of scaffolds remained consistently stable at ~ 80% (p = 0.998)*,* demonstrating that GO achieves high-strength enhancement while fully preserving the scaffold’s excellent toughness.*

### X-ray diffraction (XRD) analysis

**Fig. 6:**
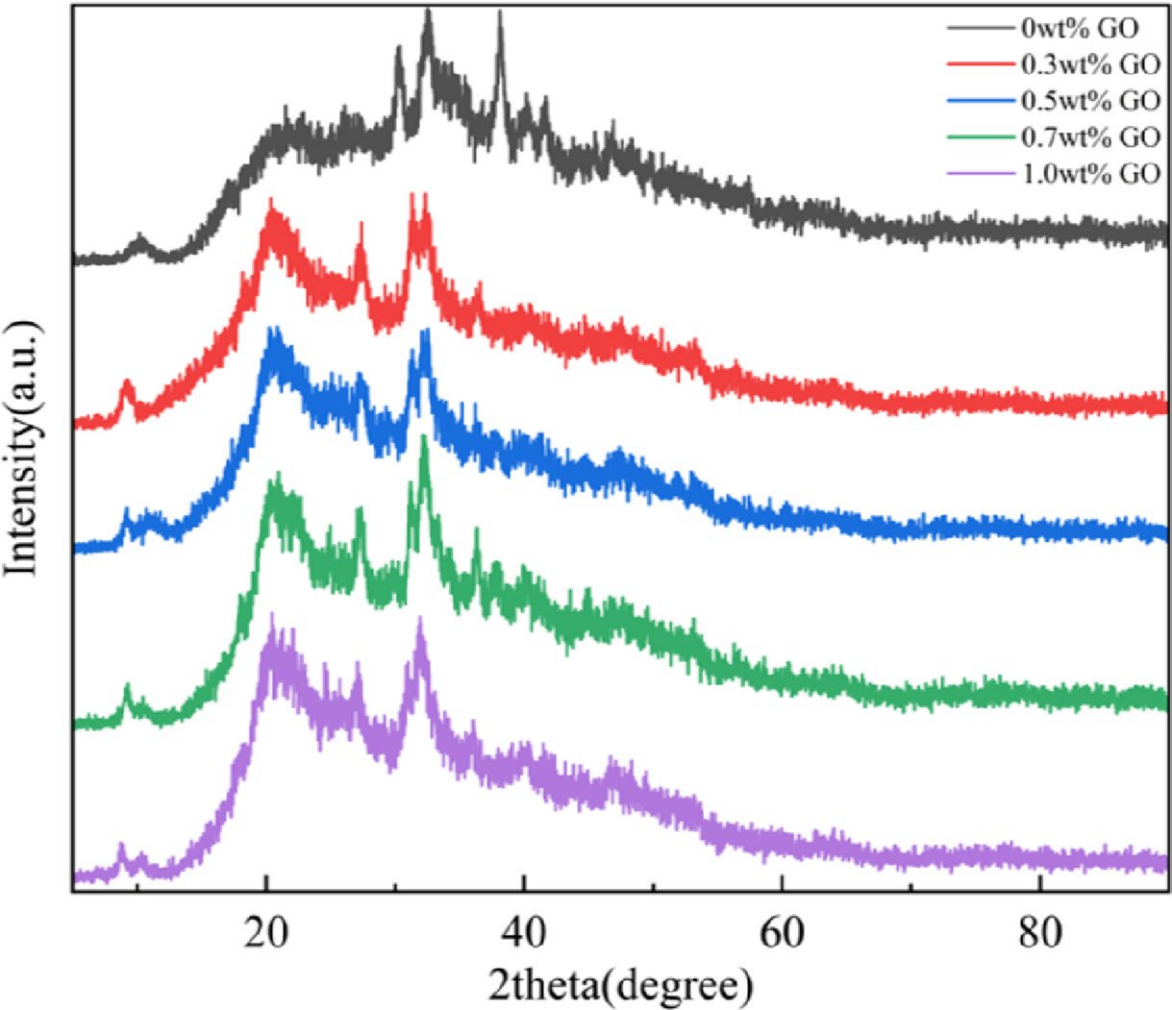
X-ray diffraction **(**XRD) analysis of CS/n-Hap/GO-x wt% scaffold crystallinity with increasing GO contents.

XRD analysis reveals the internal structural order of materials. The degree of crystallinity is determined by the ratio of the crystalline phase mass fraction to the total mass fraction of all phases present. Materials with higher crystallinity generally exhibit more ordered grain structures, which can impede dislocation movement and result in improved mechanical strength^38^. Figure 6 shows the XRD patterns of scaffolds with varying GO content. The crystallinity of scaffolds containing 1.0 wt% GO was significantly higher than that of scaffolds without GO. This enhancement is attributed to the self-assembly of GO lamellae via van der Waals forces and hydrogen bonding, forming ordered stacking structures on the CS/n-Hap surface. As GO content increased, crystallinity increased correspondingly, consistent with the experimental results. These findings further support the mechanical test results, confirming that CS/n-Hap/GO-x wt% scaffolds exhibited optimal mechanical performance at 1.0 wt% GO.

### Physical structure of CS/n-Hap/GO-x wt% scaffolds

**Fig. 7:**
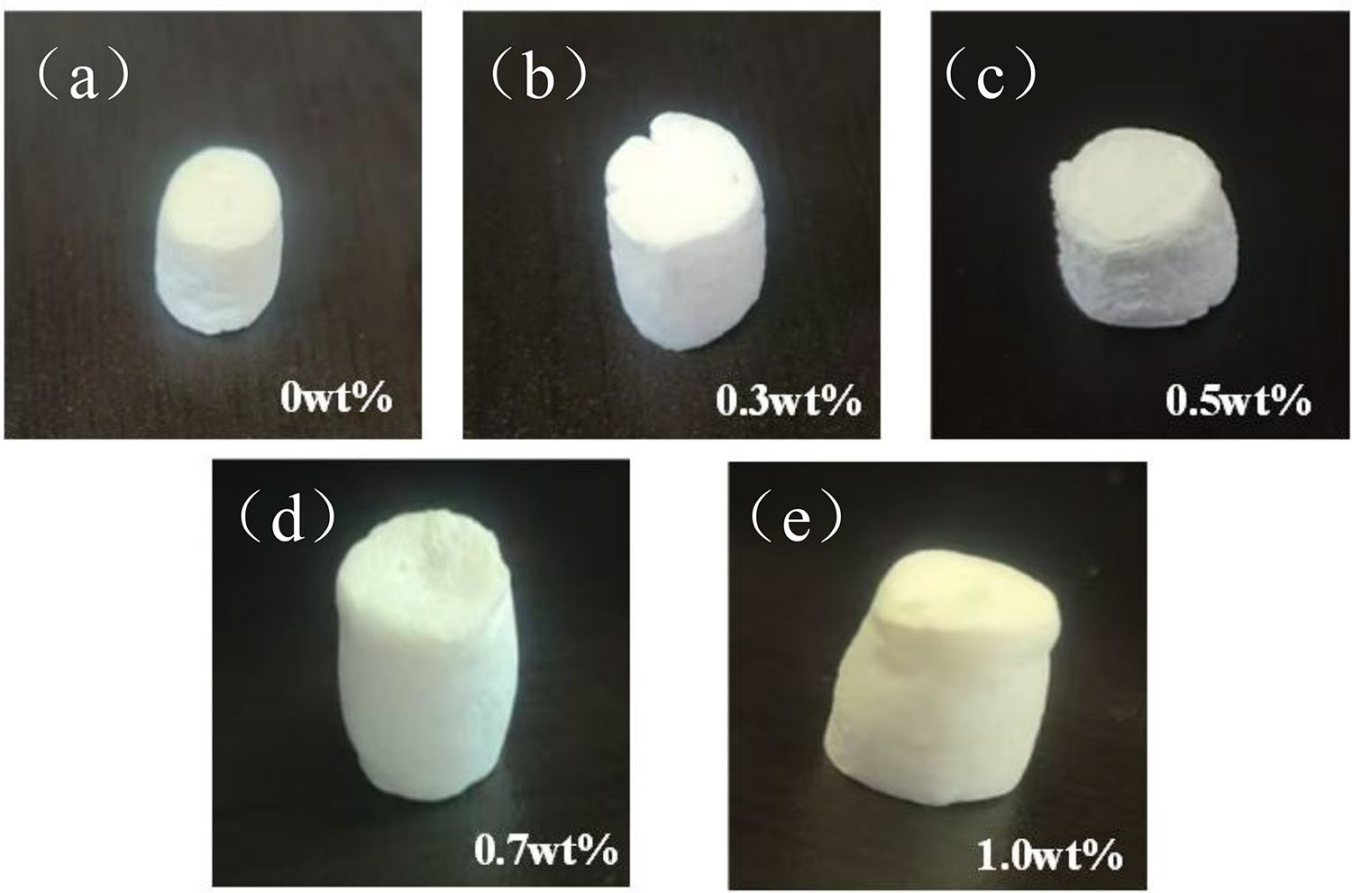
Images showing the physical structure of scaffolds with increasing GO contents. (**a**) scaffold material with 0 wt% GO; (**b**) scaffold material with 3 wt% GO; (**c**) scaffold material with 5 wt% GO; (**d**) scaffold material with 7 wt% GO; (**e**) scaffold material with 10 wt% GO.

The physical appearance of scaffold materials partially determines their potential applications. Figure 7 shows the macroscopic appearance of scaffolds with different percentages of GO contents. Scaffolds without GO appeared as white, sponge-like materials,, whereas GO incorporation gradually darkened the color. Higher GO content resulted in darker color due to the black appearance of GO suspensions after ultrasonic dispersion. At room temperature, the scaffolds exhibited a columnar, sponge-like structure with moderate plasticity, allowing shape modification using different freeze-drying molds. The scaffolds were compressible, and their shape could be adjusted for various applications by swelling within appropriately shaped molds.

### Porosity and swelling ratio

**Fig. 8:**
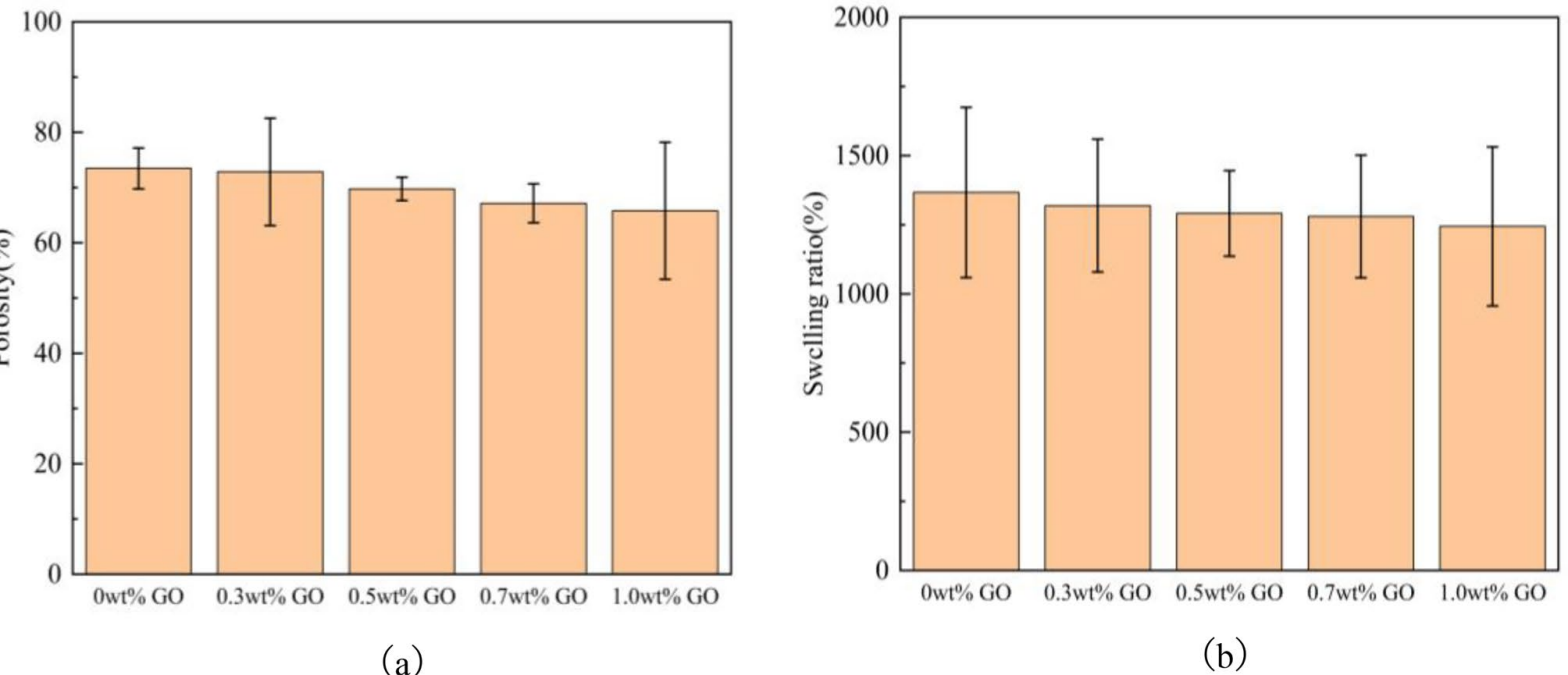
Porosity (**a**) and swelling ratio (**b**) of CS/n-Hap/GO-x wt% scaffolds with different GO contents.

Porosity and swelling ratio are key parameters for evaluating scaffold structure. Appropriate porosity is essential for nutrient transport, cell migration, proliferation, and metabolite exchange^37,38^. Figure 8 shows the porosity and swelling ratio of CS/n-Hap/GO-x wt% scaffolds. Porosity decreased gradually with increasing GO content (Fig. 8a). As shown in Table 4, even at 1.0 wt% GO, the scaffold porosity remained at approximately 65%, which is suitable for cell migration, growth, and metabolic activity, making it appropriate for bone repair applications.

**Table 4:** Porosity of CS/n-Hap/GO-x wt% scaffolds with different GO contents.

CS/*n*-Hap/GO-x wt%	0 wt%	0.3 wt%	0.5 wt%	0.7 wt%	1.0 wt%
Porosity	73.5%	72.4%	69.8%r	67.1%	65.8%

The swelling ratio reflects scaffold hydrophilicity and plays an important role in nutrient transport^39,40^. Figure 8b shows that the lowest swelling ratio (1244.1%) was observed at 1.0 wt% GO. Increased GO content promoted physical cross-linking with CS/n-Hap, resulting in a denser structure that restricted water penetration and reduced solubilization. Excessive swelling can lead to pore enlargement, structural collapse, reduced mechanical strength, accelerated degradation, and cell detachment. Thus, GO incorporation preserved scaffold hydrophilicity while effectively reducing swelling, enabling efficient nutrient transport without compromising structural integrity.

Overall, with increasing GO content, porosity and swelling ratio decreased slightly, while mechanical performance improved. Considering all factors, optimal scaffold performance was achieved at 1.0 wt% GO.

### Degradation rate

The degradation rate describes the rate of material decomposition in vivo or in a specific in vitro environment and depends on interactions between the material and its microenvironment. In this study, the analysis was performed using the average values obtained from three independent degradation tests. Figure 5shows the in vitro degradation behavior of scaffolds with varying GO content. Scaffolds without GO exhibited the highest mass loss (53.2%) after 21 days. The degradation rate of this scaffold is approximately 10% lower than that of similar composite scaffolds developed by Mohandes et al.^41^ over the same time period with a continued trend toward degradation. GO incorporation significantly reduced degradation rate, maintaining scaffold stability through day 21. All GO-containing scaffolds exhibited degradation rates of approximately 40% or lower, meeting basic requirements for bone scaffolds. The lowest degradation rate (33.8%) was observed at 1.0 wt% GO.

The degradation process can be divided into three stages: initial, deep, and final degradation^42^. During the initial stage, PBS contacted the scaffold surface without altering CS chemical structure, and pH remained stable. In the deep degradation stage, PBS penetrated amorphous CS regions, breaking –COOH and –OH groups and reducing solution pH. In the final stage, CS was decomposed into CO₂ and H₂O, and pH remained low. However, n-Hap incorporation stabilized pH changes by releasing alkaline ions that neutralized acidic degradation products. GO attachment further increased scaffold density, slowing degradation and enhancing stability.

*Regarding degradation behavior*,* in vitro experiments demonstrated that GO also exhibited a significant dose-dependent delaying effect on degradation rates. After 21 days of simulated body fluid immersion*,* the cumulative mass loss rate of 1.0 wt% GO scaffolds was only (6.13 ± 0.22)%*, significantly lower than that of the 0 wt% GO control group (13.09 ± 0.16)% (*p < 0.001),* representing a 53.2% reduction in degradation rate. Through kinetic fitting of the degradation curves*,* the apparent degradation rate constant k was estimated to decrease by approximately 60%. These results quantitatively confirm that GO*,* as a two-dimensional nanobarrier*,* effectively inhibits the erosion of water and ions*,* thereby extending the in vitro service life of the scaffolds by more than twofold.*

*As shown in* Fig. 5*and* Table 3, *the data provide a quantitative demonstration of the synergistic optimization effect of GO on the mechanical strength and degradation stability of CS/n-HAP scaffolds. As the GO content increased from 0 to 1.0 wt%*,* the compressive strength of the material was nearly 6-fold enhanced*,* while the degradation rate over the same period (21 days) was reduced by more than half. This simultaneous achievement in strength improvement and degradation reduction makes good progress in material design in this study.*

### Effect of scaffold material on cell survival

**Fig. 9:**
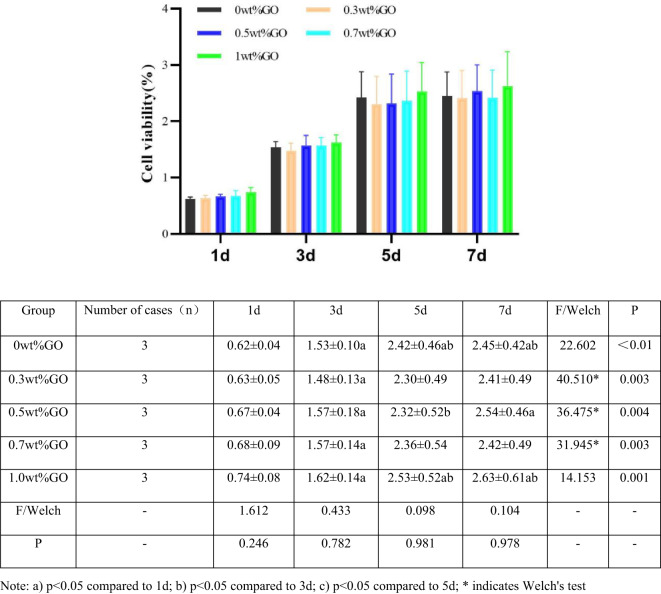
Effect of CS/n-Hap/GO-x wt% scaffolds with different GO contents on MC3T3-E1 cell proliferation at 1, 3, 5, and 7 days (*n* = 3). All measurement indicators in this study were quantitative data, which followed normal or nearly normal distribution. Descriptive statistics were employed, with one-way ANOVA used for pairwise comparisons between multiple groups and LSD for comparisons between groups. If variances were not homogeneous, Welch’s test was applied. Games-Howell test was used for comparisons between multiple groups. The significance level was set at α = 0.05.

Currently, the CCK-8 assay is widely used to evaluate in vitro cytotoxicity by measuring mitochondrial dehydrogenase activity^42,43^. The WST-8 substrate is reduced to an orange-yellow formazan, and its absorbance at 450 nm correlates positively with the number of viable cells (Fig. 9). Regarding the time effect (longitudinal comparison), the absorbance (OD) values of MC3T3-E1 cells on all five scaffold groups (0GO to 1.0GO) showed sustained and significant growth from day 1 to day 7. Statistical annotations within each group (a, b) demonstrated that the values on days 3, 5, and 7 were significantly higher than those on day 1 (*p* < 0.05), with some groups also showing significant increases on days 5 and 7 compared to day 3. These results confirm that cells could undergo normal logarithmic proliferation on all material surfaces without growth inhibition. Regarding inter-group effects (cross-sectional comparison), at each identical time point (days 1, 3, 5, and 7), there were no statistically significant differences in cell OD values between groups with different GO content (all P values: 0.246,0.782,0.981,0.978, all > 0.05). Notably, even in the 1.0 wt% GO group with a magnitude leap in mechanical properties (243.41 MPa), there was no significant difference in cell proliferation activity at any time point compared with the 0 wt% control group. Overall, the addition of GO in the experimental concentration range (0–1.0 wt%) had no negative effect on the early adhesion and proliferation of MC3T3-E1 cells, which proved that the basic cell compatibility of the scaffold series was good and independent of the content of GO. On day 1, scaffolds containing 0.5 wt%, and 1.0 wt% GO exhibited higher cell viability than scaffolds without GO, with the highest viability observed at 1.0 wt% GO. On days 3, 5, and 7, all GO-containing scaffolds showed significantly higher cell proliferation, again with the highest values in the 1.0 wt% GO group. Cell viability increased proportionally with GO content, indicating that GO was non-toxic and capable of promoting cell growth. The CCK-8 assay for evaluating cell compatibility in this study was conducted in triplicate. All measurement indicators in this study were quantitative data and followed normal or nearly normal distribution. Descriptive statistics were employed, with one-way ANOVA used for comparisons between multiple groups, and the LSD test applied for inter-group comparisons. If variances were not homogeneous, Welch’s test was applied. Games-Howell test was used for comparisons between multiple groups. The significance level was set at α = 0.05. According to Tables 2 and 5, the scaffolds were classified as Grade 0 toxicity, with cell viability approaching or exceeding 100%, demonstrating excellent cytocompatibility.

**Table 5:** Cell viability and cytotoxicity grades of MC3T3-E1 cells cultured at different time points with CS/n-Hap/GO-x wt% scaffolds with different GO contents.

Groups (wt% GO)	Relative value added (%)				Toxicity grade			
	Day 1	Day 3	Day 5	Day 7	d1	d3	d5	d7
0	100	100.39	101.02	102.64	0	0	0	0
0.3	100.92	101.55	102.93	104.57	0	0	0	0
0.5	102.78	103.35	106.17	110.01	0	0	0	0
0.7	106.79	108.02	111.49	115.24	0	0	0	0
1	111.37	118.02	121.53	132.68	0	0	0	0

### Schematic of scaffold preparation mechanism

**Fig. 10:**
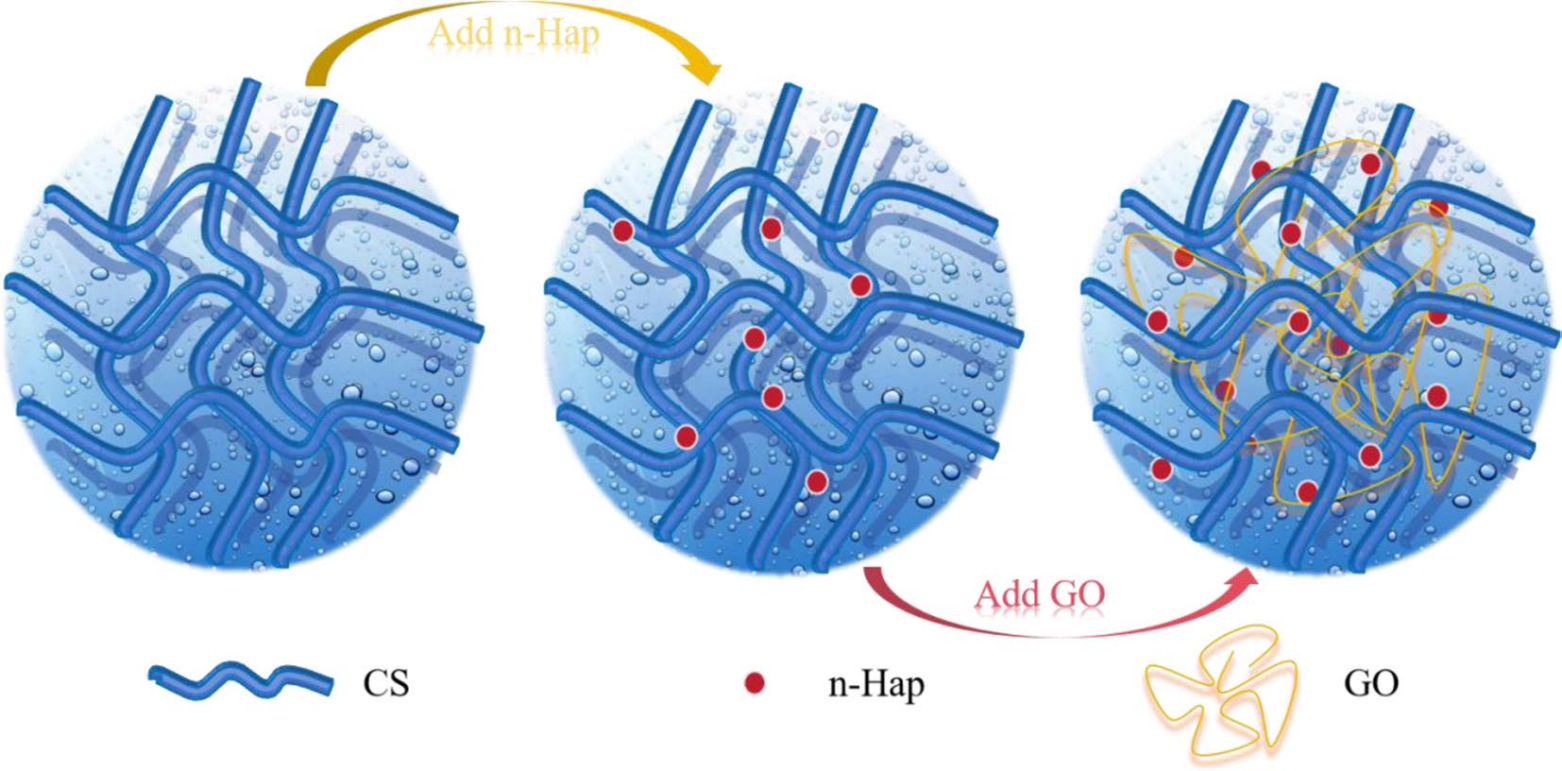
Schematic illustration of CS/n-Hap/GO-x wt% scaffold preparation.

GO is not an inert filler; rather, its unique molecular structure is the source of its multifunctional properties. It possesses a two-dimensional nanosheet structure, which provides a large specific surface area and high aspect ratio, forming the geometric foundation for the construction of physical barriers and reinforcing networks. The surface features abundant functional groups, including oxygen-containing groups such as-COOH, -OH, and C-O-C on the edges and substrate, which confer both hydrophilicity and high chemical reactivity. GO exhibits an adjustable electronic structure and surface charge, carrying a negative charge in water and its conductivity and chemical activity can be regulated by the degree of reduction. Figure 10 shows the proposed formation mechanism of CS/n-Hap/GO scaffolds. The incorporation of GO material enhances the scaffold’s strength and enables it to adsorb more n-Hap, reducing internal porosity and increasing overall material density, thereby improving stability. Simultaneously, the oxygen-containing functional groups on the GO surface exhibit excellent hydrophilicity, allowing it to adsorb large amounts of proteins and promote tissue regeneration. As the GO content increases, the biocompatibility of the scaffold is further improved. The molecular mechanisms underlying the enhancement of mechanical stability can be attributed to a multi-level interfacial toughening effect. At the GO-CS interface, strong interfacial interactions provide the molecular basis for improved mechanical stability. At the GO-n-HAP interface, anchoring of the inorganic n-HAP phase onto GO sheets effectively prevents stress concentration, enhancing composite integrity. At the GO-GO interface, when the GO content reaches the percolation threshold (approximately 1.0 wt%), continuous three-dimensional networks are formed between layers, enabling efficient load transfer through the GO framework and inducing a nonlinear stress increase from 66.6 MPa to 243.4 MPa. An energy dissipation mechanism further contributes to mechanical reinforcement. Hydrogen and ionic bonds between GO and CS are dynamically reversible. Under external forces, these bonds can break and recombine, absorbing substantial energy. This process maintains high fracture strain (~ 80%) while improving strength, effectively preventing material embrittlement. The enhanced degradation stability of the scaffold is governed by multiple molecular pathways. First, physical barrier formation and labyrinth effect arise from the oriented arrangement of two-dimensional GO sheets within the CS matrix, particularly during freeze-drying, which creates tortuous diffusion pathways for water molecules, H⁺, and OH⁻ ions, significantly prolonging the permeation time. As a result, the degradation rate decreases linearly with increasing GO content, decreasing from 13.1% to 6.1% over 21 days, directly demonstrating the effectiveness of this barrier effect. Second, chemical crosslinking and hydrolysis inhibition play important roles. Covalent crosslinking sites may form between the- COOH groups of GO and the -NH₂ groups of CS, increasing the crosslinking density of the polymer network. Furthermore, GO sheets cover and protect the glycosidic bonds and n-HAP surface active sites on the CS chains that are susceptible to hydrolysis, thereby slowing matrix erosion. Third, microenvironmental pH buffering contributes to degradation regulation, as acidic carboxyl (-COOH) and basic hydroxyl (-OH) groups on the GO surface buffer pH fluctuations, mitigating acid-catalyzed CS chain scission.

The molecular basis for maintaining and enhancing biocompatibility is multifactorial. Toxicity avoidance and benign surface reconstruction are achieved through active site masking, whereby the sharp edges of GO and potentially oxidative active sites are encapsulated by CS and n-HAp, substantially reducing direct cytotoxicity. This mechanism explains why cell proliferation remained comparable to the control group even at high GO content (1.0 wt%).

In addition, surface energy and hydrophilicity are optimized by the introduction of GO, which modulates the hydrophilic-hydrophobic balance and energy of the scaffold surface, facilitating favorable adsorption of serum proteins (e.g., fibronectin) and creating a more conducive interface for cell adhesion. Furthermore, GO-induced nanoscale roughness and topographical patterns provide cell-guiding and nano-topological cues, mimicking aspects of the extracellular matrix and promoting cell spreading and migration. Finally, the intrinsic conductivity of GO may contribute to enhanced osteogenic performance, potentially facilitating electrical signal transmission between osteoblasts and promoting their functional differentiation.

## Summary & conclusion

Chitosan, a natural biomass-derived product, and nanohydroxyapatite, which is structurally similar to the mineral phase of human bone, were selected to prepare CS/n-Hap scaffolds suitable for bone repair applications. GO was incorporated to enhance mechanical performance, and the resulting CS/n-Hap/GO scaffolds exhibited favorable mechanical properties and good biocompatibility, demonstrating their potential for repairing damaged bone tissue. After optimizing the scaffold fabrication process, the composite materials were characterized by Fourier transform infrared spectroscopy and scanning electron microscopy, revealing a well-developed porous structure. The results also showed that the characteristic spectral features of the composites became more pronounced with increasing GO content. The effects of different GO mass fractions on CS/n-Hap/GO-x wt% scaffolds were systematically investigated to identify the formulation with optimal performance. The CS/n-Hap scaffold containing 1.0 wt% GO exhibited a stable structure, confirming the successful fabrication of CS/n-Hap/GO-x wt% composites. The porosity and swelling ratio results showed that at 1.0 wt% GO, the scaffold exhibited the lowest porosity (approximately 65%) and swelling ratio (1244.1%), indicating effective structural densification.

This study achieved a remarkable enhancement in the compressive strength of CS/n-HAP-based scaffolds, increasing from 41 MPa to 243 MPa (an increase of 493%), by incorporating GO, particularly at a critical content of 1.0 wt%. Simultaneously, the scaffolds maintained approximately 80% high strain, demonstrating that ultra-high strength was achieved without reduce toughness. The incorporation of GO successfully transformed the inherent limitation of relatively rapid degradation of the CS-based materials into slow and controllable degradation. The degradation rate decreased by 53% (from 13.1% to 6.1%) within 21 days, reaching levels comparable to those of certain slowly degrading synthetic polymers (e.g., PLLA) during the early degradation stage. Importantly, this degradation behavior preserved continuous material resorption without stagnation, achieving a good balance between structural persistence and degradability.

CCK-8 assays further confirmed that the addition of GO significantly improved mechanical strength (493% increase) and markedly delayed degradation (53% reduction) without compromising the material’s fundamental cellular compatibility. Even in the high-performance 1.0 wt% GO group, cellular proliferation behavior was indistinguishable from the control group. This achievement realized the simultaneous acquisition of high strength, slow degradation, and good compatibility in this study.

By integrating these three performance dimensions, the CS/n-HAp/GO composite scaffold exhibits a good synergy in functional properties. This study successfully overcame the common trade-off dilemma between strength, degradation, and biocompatibility in the design of bone repair materials. By introducing GO as a multifunctional nanocomponent, a progress in scaffold performance was achieved within a single material system, characterized by cortical bone–level strength (243 MPa), optimized degradation behavior (6.1% over 21 days), and preserved cytocompatibility with normal cell proliferation and no detectable toxicity. Compared with most reported natural polymer scaffolds, the CS/n-HAp/GO system demonstrates improvement in both mechanical strength and degradation stability while maintaining excellent biocompatibility. Relative to synthetic polymer scaffolds, this material achieves comparable degradation controllability while offering good biocompatibility and higher initial mechanical strength. Furthermore, in contrast to systems relying solely on GO reinforcement, the combined use of CS and n-HAp effectively mitigates potential biological risks associated with GO, enabling safe and efficient performance enhancement.

From a mechanistic perspective, a threshold GO content of approximately 1.0 wt% was identified, beyond which the scaffold exhibits a nonlinear enhancement in performance while simultaneously satisfying the requirements of high strength, slow degradation, and good compatibility. In this system, GO functions as a multifunctional component, acting concurrently as a nanoscale reinforcing scaffold, a diffusion barrier regulating degradation, and an interfacial bioactive hub. The strong intermolecular interactions among GO, CS, and n-HAp constitute the fundamental mechanism underlying the observed synergistic performance enhancement.

With regards to application prospects, this scaffold, particularly the 1.0 wt% GO formulation, demonstrates good development as a load-bearing bone defect repair material owing to its mechanical strength comparable to cortical bone, a degradation rate compatible with the bone regeneration cycle, and validated cellular compatibility. Moreover, this study provides a novel design paradigm for the development of bone repair materials.

In conclusion, the CS/n-HAP/GO composite scaffold developed in this study achieved better performance in mechanical, degradation, and biological properties compared with previously reported similar materials, as well as synergistic optimization of all three aspects. This formulation of three biocompatible materials provides a promising basis for fundamental bio-scaffold research and potential applications in bone repair.

However, there are still some limitations with this study. For example, it was not possible to thoroughly investigate how the pH control at different stages of the experiment affects the performance of the scaffolds to varying degrees. Additionally, the effects of adding scaffolds with a GO content greater than 1 wt% has not been studied. Therefore, in future research, these aspects need to be explored for further optimizing bone scaffold materials comprehensively.

## Data Availability

The data in this study are available from the corresponding author upon reasonable request.

## Abbreviations

CS: Chitosan
n-Hap: Nano-hydroxyapatite
GO: Graphene oxide
PBS: Phosphate-buffered saline
CCK-8: Cell Counting Kit-8
SEM: Scanning Electron Microscope
XRD: X-ray Diffraction Performance Test
XPS: X-ray Photoelectron Spectroscopy
FTIR: Fourier Transform Infrared Spectroscopy
